# Supplementary material for: The causal relationship between the human gut microbiota and pyogenic arthritis: a Mendelian randomization study
Source: Front Cell Infect Microbiol. 2024 Nov 26;14:1452480. doi: 10.3389/fcimb.2024.1452480 (PMC11629706; doi:10.3389/fcimb.2024.1452480)
Supplement: Supplementary file 1 [file DataSheet1.zip › mendelian test/ebi-a-GCST90027554.csv_forest.pdf]

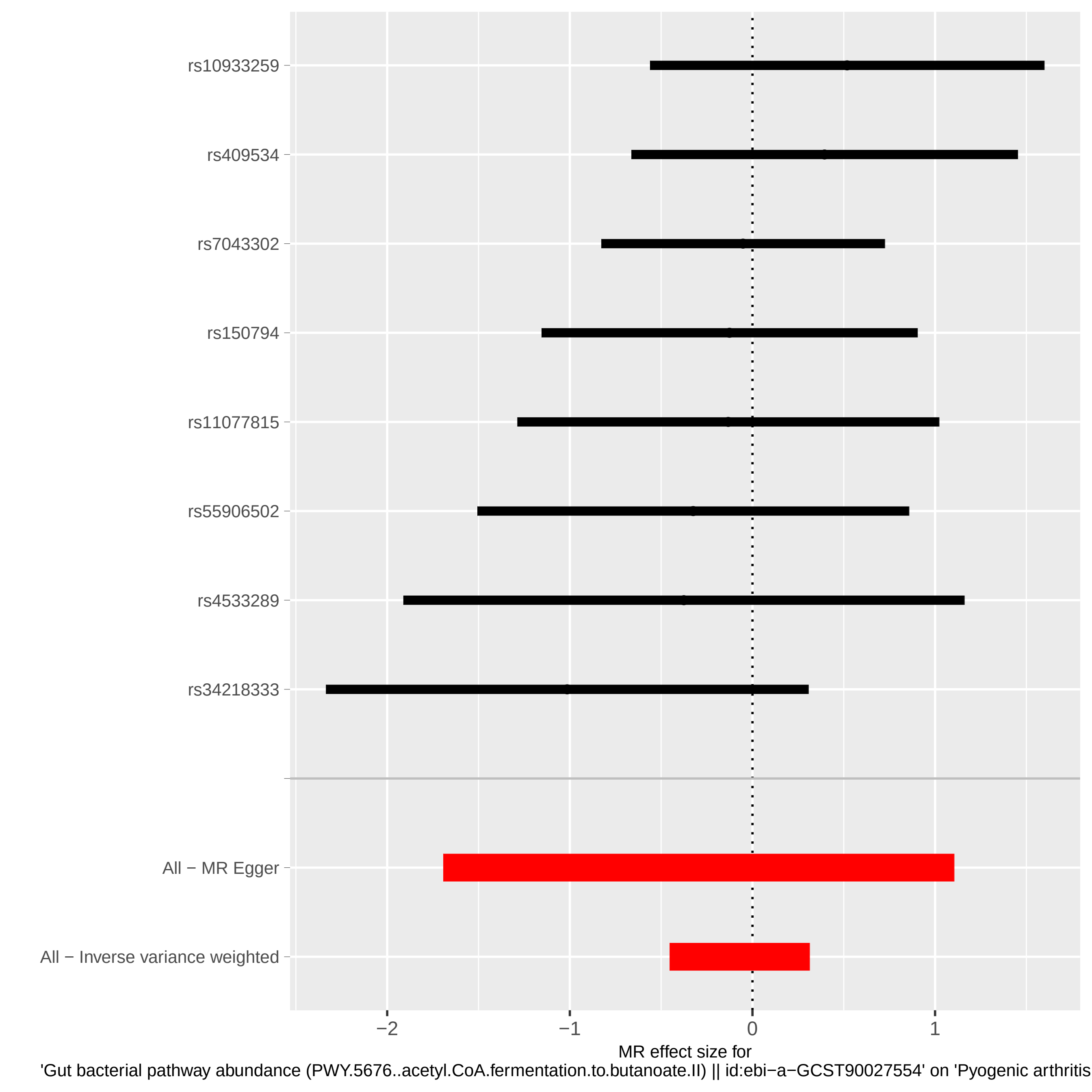
rs10933259
rs409534
rs7043302
rs150794
rs11077815
rs55906502
rs4533289
rs34218333
All − MR Egger
All − Inverse variance weighted
−2
−1
0
1
MR effect size for
'Gut bacterial pathway abundance (PWY.5676..acetyl.CoA.fermentation.to.butanoate.II) || id:ebi−a−GCST90027554' on 'Pyogenic arthritis
